# Understanding the Landscape of Multi-Cancer Detection Tests: The Current Data and Clinical Considerations

**DOI:** 10.3390/life14070896

**Published:** 2024-07-19

**Authors:** Cody E. Cotner, Elizabeth O’Donnell

**Affiliations:** 1 Department of Medicine, Brigham and Women’s Hospital, Boston, MA 02115, USA; ccotner@mgb.org; 2 Harvard Medical School, Boston, MA 02115, USA; 3 Department of Medical Oncology, Dana-Farber Cancer Institute, 450 Brookline Ave. Boston, Boston, MA 02115, USA

**Keywords:** multi-cancer detection tests, cancer screening, Galleri, CancerSeek

## Abstract

Multi-cancer detection (MCD) tests are blood-based assays that screen for multiple cancers concurrently and offer a promising approach to improve early cancer detection and screening uptake. To date, there have been two prospective interventional studies evaluating MCD tests as a screening tool in human subjects. No MCD tests are currently approved by the FDA, but there is one commercially available MCD test. Ongoing trials continue to assess the efficacy, safety, and cost implications of MCD tests. In this review, we discuss the performance of CancerSEEK and Galleri, two leading MCD platforms, and discuss the clinical consideration for the broader application of this new technology.

## 1. Introduction

Universally, cancer is a leading cause of morbidity and mortality [1,2]. In the United States, it is the second leading cause of death and is projected to claim the lives of 611,720 Americans in 2024 [3]. Not only is cancer responsible for enormous human costs but also substantial financial costs: the United States spends approximately USD 200 billion per year on cancer care, and patients spend USD 20 billion yearly in out-of-pocket costs [4,5].

Due to reduced smoking and tobacco use, improved treatments, and cancer-specific screening initiatives, cancer mortality in the United States has declined by approximately 33% between 1991 and 2021 [3]. Improving screening initiatives is a compelling strategy to further reduce cancer mortality, since nearly all cancer-attributable deaths occur in patients who have cancers that have metastasized and are in the advanced stages [6,7]. Most cancers evolve from pre-malignant lesions caused by genetic alterations that induce the monoclonal expansion of cells that continue to accumulate mutations and eventually become malignant [8]. Further alterations confer invasive and metastatic ability for a subset of cells in the primary tumor [9]. For many cancers, the tumorigenesis process takes decades and, therefore, there are multiple opportunities for screening across this continuum [10].

Cancer screening initiatives aim to screen at-risk asymptomatic populations for precancerous or early-stage malignancies to reduce cancer-associated mortality. When cancer is detected at early stages, treatment is more effective and the prognosis improves for nearly all cancers [10,11]. Furthermore, the costs associated with caring for patients with cancer increase with cancer stage [12]. For example, it costs USD 7640 to care for patients with stage I prostate cancer compared to USD 58,783 for patients with metastatic disease [13].

The United States Preventive Services Task Force (USPSTF) currently advises breast, cervical, colorectal, and lung cancer-specific screening interventions for the at-risk members of the general population. These screening programs have been shown to reduce cancer-specific mortality [14,15,16,17]. Yet, these four malignancies are predicted to be responsible for 711,930 (35.6%) of the 2,001,140 new cancer cases and 224,690 (36.7%) of the 611,720 cancer deaths in 2024 [11,18]. Furthermore, adherence to select existing screening programs is poor: for example, less than 60% of eligible patients undergo lung cancer screening with a low-dose CT scan [19].

There remains substantial unmet need for early cancer detection platforms that increase the number of detectable cancers and improve already existing screening methods. The sampling of body fluids—known as liquid biopsies—to detect the presence of cancer has generated substantial interest in recent years [10,20]. Due to advances in genomics and machine learning, blood-based testing may be used to screen for multiple cancers at once.

## 2. Multi-Cancer Detection Testing

**Figure 1:**
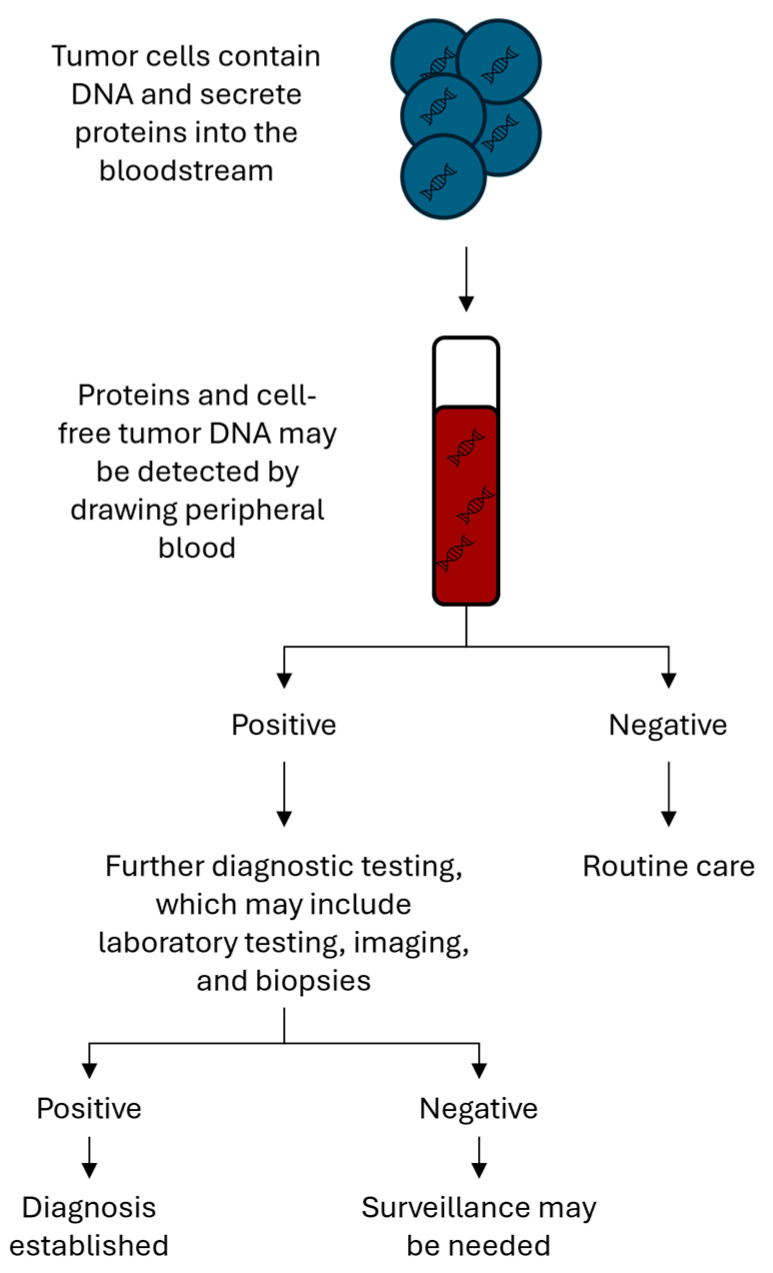
Testing algorithm for multi-cancer detection tests. Peripheral blood is used to screen for biomarkers that indicate the presence of a malignancy. If positive, patients will require further testing. Research is ongoing to determine whether patients with a positive screening test require ongoing surveillance.

Multi-cancer detection (MCD) tests are blood assays that screen for cancer-specific biomarkers and can indicate the presence of an underlying malignancy (Figure 1). MCD tests operate on the premise that many cancers share molecular features that can be detected prior to the onset of clinical symptoms [21]. For example, circulating cell-free DNA (cfDNA) released by tumor cells during apoptosis can be detected in the blood stream, as can epigenetic modifications of the cfDNA (e.g., methylation patterns, circulating RNA) [22]. Other possible biomarkers include circulating tumor cells, exosomes, platelet-associated RNA, and proteins [23].

MCD tests are promising screening tests because they are non-invasive blood draws, theoretically able to identify patients with cancer at early, intervenable stages, and can screen for multiple cancers with one test, most of which do not have screening options available. MCD tests also enable the screening of less common cancers, for which dedicated screening tests are not cost-effective or practical [18]. By screening for many cancers at once, the number needed to screen to find one person with a malignancy is much lower and makes screening for uncommon cancers more attainable. However, because MCD tests are screening and not diagnostic tests, a positive test will lead to further confirmatory and possibly invasive testing [24]. Therefore, the test characteristics, particularly the specificity and positive predictive value, are important when evaluating these tests to determine the anticipated number of false positive cases [25].

There are numerous MCD tests in the pipeline, most of which are in the early developmental stages [23]. MCD platforms are usually developed in case-control studies that involve recruiting cohorts of patients with previously diagnosed cancer and controls without known cancer. These data can then be used to develop and validate assays and machine learning algorithms that can identify patients with cancer from healthy controls [26]. Galleri produced by GRAIL (Menlo Park, CA, USA) and CancerSEEK, and its successor CancerGuard, produced by ExactSciences (Madison, WI, USA) are the most clinically advanced testing platforms and have each conducted large, prospective cohort studies evaluating each test’s screening ability. Here, we review the clinical data available from the Galleri and CancerSEEK studies and discuss the considerations related to widespread MCD adoption.

## 3. CancerSEEK

The CancerSEEK test uses mutation data from cfDNA and protein biomarkers to identify a cancer signal in the blood stream. CancerSEEK evaluates for mutations in 61 regions of 16 cancer-driver genes (*AKT1*, *APC*, *BRAF*, *CDKN2A*, *CTNNB1*, *EGFR*, *FBXW7*, *FGFR2*, *GNAS*, *HRAS*, *KRAS*, *NRAS*, *PIK3CA*, *PPP2R1A*, *PTEN*, and *TP53*) and measures the levels of eight cancer-associated protein biomarkers (CA19-9, CA-125, CEA, HGF, MPO, OPN, PRL, and TIMP-1) [27]. The test can predict a cancer site of origin using cfDNA and protein biomarker data (the test uses 31 additional protein biomarkers to predict the site of origin), though most predictions rely on protein biomarker data since driver mutations are usually not tissue specific.

CancerSEEK’s validation study was published in 2018 and included 812 (45%) participants without cancer and 1005 (55%) participants with one of eight cancer types (ovarian, liver, esophageal, pancreatic, stomach, colorectal, lung, or breast cancer) who had stage I-III disease [27]. Participants with cancer were enrolled shortly after their diagnosis and excluded if they had already started disease-directed therapy (i.e., surgery or neoadjuvant chemotherapy). The specificity of the test was 99.1%, with only seven of the eight-hundred and twelve participants without known cancer having false positive results. The test returned positive for 626 of 1005 participants with cancer for an overall sensitivity of 62.3% and correctly predicted the site of origin in 62.9% of true positives. Notably, the test sensitivity varied substantially by cancer stage and type. For example, the test sensitivity was 70.2% among participants with stage III disease compared to 49.9% among participants with stage I disease. CancerSEEK performed particularly well in participants with ovarian and gastrointestinal cancers, which do not have screening tests available and may be cured with early surgery, and least well in participants with breast cancer; the sensitivity for ovarian, liver, and breast cancer was 98.1%, 97.5%, and 33.4%, respectively. Interestingly, the genetic testing of tumor tissue from 153 patients with true positive tests was performed, and investigators found the mutation detected from circulating cfDNA was identical to the mutation found in the tumor in 90% of cases which could guide precision therapies without the need for invasive diagnostics.

After CancerSEEK demonstrated high specificity and the ability to detect malignancies without available screening tests at early stages in its case-control study, its performance as a screening tool in a large population was evaluated. CancerSEEK’s DETECT-A study enrolled 10,006 women without a history of cancer from 65 to 75 years of age between September 2017 and May 2019 across 18 clinical sites in a single integrated health system [28]. The investigators chose to include only women to better evaluate the test’s ability to screen for ovarian cancer, given the high sensitivity for ovarian cancer observed in the validation study.

Participants provided a blood sample, and those with abnormal results were asked to return for repeat testing to confirm the results and exclude clonal hematopoiesis of indeterminate potential (CHIP) as a possible cause for the cancer signal by evaluating a larger amount of white blood cell DNA. If participants had a persistently positive test and CHIP excluded, then their medical records were reviewed by a committee composed of experts from several specialties to rule out possible non-cancer related causes of the abnormal results. The results were returned to the committee after an average of 7.0 months elapsed from the first blood draw. If the committee could not identify a non-cancer related cause, then participants were invited to undergo a PET scan to search for an underlying malignancy. Participants with a PET concerning for a malignancy were referred to the appropriate specialist for diagnosis and treatment. Charts were reviewed at one-year follow-up to determine if patients had been diagnosed with a malignancy.

In DETECT-A, CancerSEEK had an overall sensitivity of 23.5%, a specificity of 98.9%, and a positive predictive value of 19.4%. There were 490 (4.9%) participants who had a positive CancerSEEK test, of whom 134 (1.4%) had a positive confirmatory test. A PET-CT or additional imaging was recommended and obtained for 127 participants (116 underwent PET and 11 underwent other imaging) and concerning for a malignancy in 64 cases. Twenty-six participants were subsequently diagnosed with a malignancy, of whom eight (31%) had stage I–II disease and fourteen (54%) had a cancer with no USPSTF-advised screening available. The number needed to detect one cancer during screening was 381 and 1245 to detect one stage I-II cancer. There were one-hundred and eight participants who had false positive results, and sixty-three (58%) underwent a PET-CT, nineteen (18%) underwent a non-surgical procedure, and three (3%) underwent a surgical procedure.

## 4. Galleri

The Galleri test detects cancer-specific DNA methylation patterns covering more than 100,000 DNA regions from circulating cfDNA [29]. Since the DNA methylation of CpG dinucleotides regulates tumorigenesis and tissue differentiation, among other things, it is possible to identify methylation patterns that indicate a malignancy and predict the primary site of a malignant tumor when present [21].

Galleri’s validation study was published in 2021 and a substudy part of the larger circulating cell-free genome atlas (CCGA) study (NCT02889978) that aimed to choose the highest performing assay and subsequently train and validate it [30,31,32]. The validation substudy included 2823 (69%) participants with known cancer and 1254 (31%) without cancer [30]. In the cancer cohort, participants with over 50 cancer types and stage I–IV disease who had not yet started cancer-directed therapy were included. The test was 99.5% specific with only six false positives among one-thousand two-hundred and fifty-four participants without cancer. A cancer signal was detected in 1453 of 2823 patients with cancer for an overall sensitivity of 51.5%. When the assay returned a positive result, it correctly predicted the cancer site of origin in 88.7% of cases. Like CancerSEEK, the sensitivity of detection increased with cancer stage (stage I: 16.8%, stage II: 40.4%, stage III: 77.0%, stage IV 90.1%) and varied substantially by cancer of origin. The assay performed well for GI malignancies, while the assay was less sensitive for solid tumors originating from the breast, kidney, prostate, thyroid, and uterus.

After the completion of the validation studies, Galleri was prospectively studied in both asymptomatic and symptomatic populations. The PATHFINDER study (NCT04241796) evaluated its use as a screening tool in an asymptomatic population and enrolled 6662 men and women aged 50 years or older across seven US-based health centers between December 2019 and December 2020 [33]. Participants with a history of cancer were eligible if their cancer was treated and treatment was completed three years or more prior to enrollment. Approximately 25% of participants in PATHFINDER had a history of malignancies. The enrolled participants provided a blood sample, and test results were returned to the ordering medical team at the enrolling sites approximately two weeks after collection. The results were reported as a binary outcome (cancer signal detected or not) and, if a signal was detected, it included the predicted site of origin. The clinical site medical team, in consultation with the study investigator and interdisciplinary care team, directed all subsequent workup when the tests were positive and decided when the diagnostic workup was complete. The investigators reviewed the electronic health records 12 months after enrollment and contacted the participants if necessary to determine whether the participants had been diagnosed with a malignancy.

Galleri had a specificity of 99.1% and positive predictive value of 38%. The PATHFINDER investigators did not report the overall sensitivity due to the lack of a gold-standard diagnostic for cancer, though the test was positive in 35 of 121 patients diagnosed with a malignancy during the study period (28.9%).

There were 92 (1.4%) participants who had a positive cancer signal detected, of whom 35 (38%) were subsequently diagnosed with cancer. Six (17%) were diagnosed with recurrent cancer, twenty-eight with (80%) new cancer, and one (3%) had both. Of the 28 patients with a new cancer, 25 (86%) were diagnosed with cancers without a USPSTF-advised screening test available and 14 (50%) were diagnosed with stage I-II disease. Six of the fourteen (43%) newly diagnosed stage I–II cancers were follicular lymphomas, which are frequently indolent and have five-year overall survival rates of greater than 90%. The number needed to detect one new cancer case during the screening was 189 and 473 to detect one new cancer case with stage I-II disease. The cancer site of origin was accurately predicted in 85% of participants with true positive results. There were 57 participants with false positive results, and 50 (88%) underwent additional laboratory testing, 53 (93%) underwent imaging testing, 16 (28%) underwent a non-surgical procedure, and a single person (2%) underwent a surgical procedure. Among the fifty-three participants who underwent imaging testing, thirty-five (61%) underwent a PET-CT, twenty (35%) underwent a CT, fifteen (26%) underwent an MRI, eight had (14%) an ultrasound, and four (7%) had a mammogram.

SYMPLIFY (ISRCTN10226380) was a prospective cohort study that sought to evaluate Galleri as a diagnostic tool in patients with suspected malignancies or non-specific symptoms of malignancies and included 5461 participants. Patients were eligible for testing if they were referred for urgent investigation for a possible gynecologic (26%), lung (5%), lower gastrointestinal (19%), or upper gastrointestinal cancer (40%) based on the National Institute for Health and Care Excellence (NICE) guidelines or had non-specific symptoms meriting referral to a national health service rapid diagnostic center (9%). After enrollment, patients were followed for 9 months or until diagnostic resolution. Unlike PATHFINDER, the results were not returned to the patients or clinicians, as the aim of the study was to determine the diagnostic performance of the test in this population. The most common symptoms prompting referral for testing were unexpected weight loss (24.1%), change in bowel habits (22%), post-menopausal bleeding (16%), rectal bleeding (15.7%), and abdominal pain (14.5%).

Galleri had an overall sensitivity of 66.3%, detecting a cancer signal in 244 of the 368 participants with cancer diagnosed, and a sensitivity of 39.2% in participants with stage I-II disease. The specificity was 98.4%, and, in this population, the positive and negative predictive values were 75.5% and 97.6%, respectively. The most common symptom prompting referral was unexplained weight loss, for which the test sensitivity was 73.4%, the specificity was 98.8%, the positive predictive value was 85.1%, and the negative predictive value was 97.6%. Among the patients with true positive test results, Galleri correctly predicted the primary site in 85.2% of patients. The sensitivity and accuracy of the predicted site of origin was higher among patients with stage III–IV cancer than patients with stage I–II disease.

## 5. Regulatory Status and Ongoing Trials

Neither GRAIL nor Exact Sciences have obtained FDA approval for their tests to date, though both have received a breakthrough device designation by the FDA. The Galleri test may be prescribed by any physician as a laboratory-developed test (LDT) and is the only MCD test currently available for prescription in the United States [34]. LDTs are developed by individual laboratories that are regulated by the Centers for Medicare and Medicaid Services via the Clinical Laboratory Improvement Amendments (CLIA). However, CMS has limited oversight over the reliability or quality of LDTs [35]. LDTs are not under the regulatory oversight of the FDA and have limited coverage by insurers, though proposed legislation may cause LDTs to fall under FDA purview in the future [35,36]. Galleri is not routinely covered by insurers [37].

There has been substantial investment by both government and private entities to further evaluate MCD tests in asymptomatic populations (Table 1). The national health service Galleri trial is a prospective randomized trial that has enrolled over 140,000 people between the ages of 50 to 77 years. The primary endpoint is the incidence rate of stage III and IV cancers in the intervention compared to the control arm, with the comparison of cancer-specific mortality as one secondary endpoint [38]. The National Cancer Institute launched the Cancer Screening Research Network in January 2024 to more rigorously study the feasibility of MCD tests [39]. The Vanguard study, a pilot study that will enroll approximately 24,000 people, is the first study being planned and will be used to inform a much larger randomized trial in the United States [40].

## 6. Clinical Considerations for MCD Adoption

In PATHFINDER and DETECT-A, approximately 1% of patients had false positive tests that resulted in downstream non-invasive and invasive diagnostic testing in select patients. While the overall number of patients who underwent unnecessary testing and procedures was small in these prospective studies, a screening test applied to millions of people may result in substantial downstream testing and associated costs, complications, and anxiety which must be carefully considered. In a qualitative study funded by GRAIL that interviewed 50–77-year-old participants and explored their attitudes toward MCD testing, participants acknowledged false positive results may cause significant unnecessary anxiety and anger while others in the study considered false positive tests as necessary and acceptable if it meant cancers could be detected at an early stage [41].

All currently available screening tests are not perfectly specific, and many patients undergoing USPSTF advised screening tests will have false positive results. Breast cancer screening with mammography has a specificity of approximately 95% [42], while studies of lung cancer screening via low-dose CT scan mostly report variable specificities that are generally >75% (Table 2) [14,43,44]. All patients with a positive mammogram or CT scan are recommended to undergo further diagnostic testing, which frequently includes biopsies. In fact, approximately 7% of women screened over a 10-year period with annual mammograms are recommended to undergo biopsy based on a false positive test [45]. The positive predictive value of screening tests, defined as the probability that a patient with an abnormal test has the disease, varies depending on the prevalence of a disease in a population and is less than 10% for some USPSTF-advised screening tests in the United States. For example, the positive predictive values for mammography and low-dose CT lung cancer screening are 4.4% [46] and 3.8% [47], respectively, compared to positive predictive values of 19.4% and 38% for CancerSEEK and Galleri, respectively.

Survey-based studies have suggested that patients are highly tolerant of false positive results: in one study, 63% of women thought that 500 or more false positive results per life saved was reasonable, and 37% thought 10,000 or more would be reasonable [54]. While there seems to be a high risk tolerance of false positive results, these results can cause persistent psychological distress which should be considered in a screening test’s risk-benefit calculus [55]. The median time to diagnostic resolution may impact the degree of anxiety participants experience and was longer in PATHFINDER compared to other screening tests. For example, the time to biopsy after an abnormal screening result is approximately 23 days for breast cancer, while patients in PATHFINDER had a median time to diagnostic resolution of 79 days [56]. Notably, the PATHFINDER study was conducted during the COVID-19 pandemic which may have prolonged the time to diagnostic resolution. Nonetheless, a longer time to diagnostic resolution would be expected with MCD testing compared to traditional single-cancer screening methods, since the diagnostic pathway is not as clear. Furthermore, the appropriate follow-up duration for these tests is not clear, since positive tests may indicate an underlying malignancy that is too early to be seen on imaging or endoscopically. MCD tests should seek to study the impact of false positive results on patients currently enrolled in their studies, establish proposed diagnostic pathways that guide workup after a positive test, and clarify the follow-up procedures for patients with positive tests and negative diagnostic workups.

The clinical implementation of MCD testing should include a plan for ongoing surveillance for those individuals with a positive result and a negative diagnostic evaluation. There is currently no evidence-based guidance for the interval or duration of ongoing follow-up for false positive cases. Further, there is no guidance for patients with negative test results on when and if to re-test. The development of risk assessment tools could potentially complement the use of MCD tests in determining who would benefit from screening and at what frequency.

## 7. Equity and Access Considerations

Possible benefits of blood-based screening are the potential for expanded access and improved adherence to screening. Patients who live in medically underserved regions have less access to healthcare providers and lower cancer screening rates [57,58,59]. MCD tests may improve access to screening, since they are simple blood draws and, by themselves, do not require substantial health infrastructure [24]. However, the implementation of MCD testing without thoughtful planning has the potential to exacerbate existing disparities through multiple channels. First, a positive test may require further diagnostic testing that requires either imaging or procedures performed by specialists. Providers offering MCD testing should have accessible referral pathways for patients whose screening tests are abnormal. Second, the cost of MCD testing and subsequent workup are large barriers to adoption for economically disadvantaged populations. To expand access to broad populations, MCD tests will need to be covered by third party payers so as not to exacerbate disparities between those who can pay out-of-pocket for the tests’ costs versus those who cannot [60]. In a study assessing payers’ attitudes towards MCDs, most (74%) payers did not expect MCD tests to reduce disparities unless barriers to accessing subsequent care are reduced and Medicaid covers MCD testing [37].

It is important that research investigations enroll a representative population to ensure similar test characteristics across populations, particularly since biomarkers used by MCD tests may differ among people from varying racial and ethnic backgrounds [61,62]. The DETECT-A and PATHFINDER cohorts enrolled a population that was >90% White, and the 140,000-person Galleri trial enrolled participants who were 93.2% White [63]. While a preliminary subgroup analysis of the patients enrolled in the CCGA study showed similar test sensitivities and specificities across race, the analysis included only 278 Black, 295 Hispanic, and 75 Asian participants [64]. The upcoming Vanguard study should prioritize the enrollment of diverse populations to confirm the tests’ efficacy across historically marginalized populations for whom substantial disparities in cancer survival exist [65].

## 8. Conclusions

MCD testing offers a promising complementary strategy to improve early cancer detection. The technology has the potential to reduce barriers and expand access to screening, but realizing its full potential requires thoughtful planning among manufacturers, payers, physicians, and patients. The successful adoption of MCD testing will require clear and accessible paths for diagnostic resolution for patients who have positive results to ensure an efficient and safe diagnostic workup for the patient and reduce the cognitive and administrative burden on the ordering physician. Clear, evidence-based guidance for the ongoing surveillance for those individuals with a positive test and negative diagnostic evaluations is needed.

Further research is ongoing and needed to fully evaluate the effectiveness and safety of MCD tests. Trials should evaluate cancer-specific mortality, stage shift, the safety of downstream diagnostic pathways, and the cost-effectiveness of MCD testing. While MCD testing may reduce cancer-related costs by identifying treatable cancers in early stages, the downstream diagnostic workup among patients with true and false positives needs to be fully evaluated on a large scale. Finally, ongoing efforts are needed to determine the target population or populations for screening and the frequency with which individuals should be screened. Current USPSTF guidelines have variable recommended start and stop screening ages based on the cancer-specific epidemiology. However, MCD tests evaluate multiple cancers with differing incidences across age groups, making it challenging to establish ideal screening ages. Furthermore, the most effective screening interval presents another layer of uncertainty. Future research endeavors should focus on defining populations at risk and assessing differing screening intervals. For example, MCD testing may further enhance screening recommendations for individuals with germline mutations or extensive smoking histories.

MCD tests have tremendous potential and may represent a paradigm shift in cancer screening. Ongoing work is needed to further evaluate and thoughtfully plan implementation to ensure equity and access for patients and sustainability for ordering providers.

## Figures and Tables

**Table 1:** Ongoing and planned prospective studies and randomized controlled trials.

Study Name	Sponsor	Study Type	Population	Size	Outcomes
NHS-Galleri (ISRCTN91431511)	NHS, GRAIL	Randomized controlled trial	Adults 50–77 years of age	140,000	Primary: cancer incidence and stage at diagnosis Secondary: cancer mortality, number of follow-up procedures, number of complications and deaths from diagnostic procedures, radiation exposure, and psychologic impact of Galleri test
PATHFINDER 2 (NCT05155605)	GRAIL	Prospective cohort	Adults ≥50 years of age	35,000	Primary: test performance and number and type of invasive procedures performed in false positives Secondary: participant-reported anxiety, perceptions of Galleri, and intention to follow standard-of-care cancer screenings, radiation exposure, and diagnostic evaluation
REFLECTION (NCT05205967)	GRAIL	Prospective cohort	Adults ≥22 years of age	17,000	Primary: describe signal and cancer detection Secondary: assess the feasibility and acceptability of Galleri from the participant’s perspective and patient-reported outcomes; to assess healthcare resource utilization associated with diagnostic workups when the test result is positive
VANGUARD [39,40]	NCI	Randomized controlled trial	Preliminarily 45–70 years	24,000	Outcomes are to be defined. The study will preliminarily have three groups and evaluate two MCD assays compared to a control group. The purpose of the trial is to inform a larger randomized trial.

SUMMIT and STRIVE are additional ongoing studies seeking to evaluate Galleri in populations undergoing LDCT and mammography screening.

**Table 2:** Sensitivity and specificity of USPSTF-endorsed tests.

Screening	USPSTF Screening Recommendation	Sensitivity	Specificity
Breast	Women aged 40–74 years [48]		
	Biennial mammography [46,49]	70–87%	89–92%
Cervical	Women aged 21–65 years [50]		
	Cytology-based screening [51]	36–100% *	96–98% *
	Cotesting with cytology and HPV [51]	93.7–100% *	90–94% *
Colorectal **	Adults aged 45–75 years [52]		
	Fecal immunochemical test (FIT) [52]	64–83%	93–96%
	Stool DNA-FIT test [52]	87–100%	84–86%
	CT colonography [52]	86–100%	Not reported
Lung	Adults aged 50–80 years with a 20 pack-year smoking history currently smoking or who quit in last 15 years [53]		
	Low-dose CT screening [47]	91–96%	73–74%
CancerSEEK	N/A	23.5%	98.9%
Galleri	N/A	28.9%	99.1%

* Sensitivity and specificity of detecting cervical intraepithelial neoplasia 3 or greater. ** Colonoscopy serves as the reference group for sensitivities and specificities of listed colorectal cancer screening tests.

## Data Availability

Not applicable.
